# Total-to-ionized calcium ratio predicts mortality in continuous renal replacement therapy with citrate anticoagulation in critically ill patients

**DOI:** 10.1186/cc11363

**Published:** 2012-05-29

**Authors:** Andreas Link, Matthias Klingele, Timo Speer, Ranja Rbah, Janine Pöss, Anne Lerner-Gräber, Danilo Fliser, Michael Böhm

**Affiliations:** 1 Klinik für Innere Medizin III, Universitätsklinikum des Saarlandes, Kirrberger Strasse, D-66421 Homburg/Saar, Germany; 2 Klinik für Innere Medizin IV, Universitätsklinikum des Saarlandes, Kirrberger Strasse, D-66421 Homburg/Saar, Germany

## Abstract

**Introduction:**

Regional citrate anticoagulation is safe, feasible and increasingly used in critically ill patients on continuous renal replacement therapy (CRRT). However, in patients with hepatic or multi-organ dysfunction, citrate accumulation may lead to an imbalance of calcium homeostasis. The study aimed at evaluating the incidence and prognostic relevance of an increased total to ionized calcium ratio (T/I Ca^2+ ^ratio) and its association to hepatic dysfunction.

**Methods:**

We performed a prospective observational study on n = 208 critically ill patients with acute kidney injury (AKI) and necessity for CRRT with regional citrate anticoagulation (CRRT-citrate) between September 2009 and September 2011. Critical illness was estimated by Simplified Acute Physiology Score II; hepatic function was measured with indocyanine green plasma disappearance rate. After achieving a steady state of calcium homeostasis patients were classified into tertiles according to the T/I Ca^2+ ^ratio (<2.0 versus 2.0 - 2.39 versus ≥2.4).

**Results:**

The T/I Ca^2+ ^ratio was determined as an independent predictor for 28-day mortality in critically ill patients with AKI on CRRT-citrate confirmed by receiver operating characteristics and multivariate analysis (Area under the curve 0.94 ± 0.02; p<0.001). A T/I Ca^2+ ^ratio ≥2.4 independently predicted a 33.5-fold (p<0.001) increase in 28-day mortality-rate. There was a significant correlation between the T/I Ca^2+ ^ratio and the hepatic clearance (p<0.001) and the severity of critical illness (p<0.001). The efficacy and safety of citrate anticoagulation, determined by blood urea nitrogen, mean filter patency and bleeding episodes, were not significantly different between the tertiles.

**Conclusions:**

In patients on CRRT-citrate T/I Ca^2+ ^ratio is closely related to the clinical outcome and emerged as an independent predictor of 28-day mortality. Larger studies are required to define the cut-off and predictive value for the T/I Ca^2+ ^ratio. This ratio is associated with hepatic and/or multi-organ dysfunction and therefore an important therapeutic target.

## Introduction

Acute kidney injury (AKI) has an incidence of 30% in intensive care units (ICUs) [1] and most frequently occurs in multiple organ dysfunction syndrome (MODS) [2,3]. Even though available studies do not demonstrate a reduction in mortality under continuous renal replacement therapy (CRRT) compared with intermittent hemodialysis [4,5], CRRT has some advantages like slow and balanced fluid removal leading to minimal variability of plasma osmolality and electrolyte disturbances with better cardiovascular and hemodynamic tolerability [6]. The main disadvantage of CRRT is the requirement for anticoagulation to prevent clotting of the extracorporeal circuit, and severe bleeding has been reported in up to 30% of these patients, with heparin being the most commonly used anticoagulant [7,8].

Regional citrate anticoagulation is an effective and safe alternative to heparin [7,9-11]. Citrate is infused into the extracorporeal circuit and chelates ionized calcium, thereby inhibiting coagulation. Citrate and chelated calcium enter the dialysate and are removed from the hemocircuit with calcium chloride (CaCl_2_) infused systemically, replacing the losses of calcium. Citrate not dialyzed through the filters enters the systemic circulation of the patient and is metabolized to bicarbonate mainly by the liver. Non-metabolized citrate chelates ionized calcium, leading to a decrease in its concentrations [11-14]. CaCl_2 _is continuously administered to achieve a steady state between citrate administration by central infusion and citrate elimination determined by liver metabolism. Once a steady-state citrate concentration is achieved, a normal ionized calcium concentration can be achieved by an increased total calcium concentration because a fraction of the ionized calcium is chelated by circulating systemic citrate. The total-to-ionized calcium ratio (T/I Ca^2+ ^ratio) should be directly proportional to the concentration of serum citrate [15,16]. Therefore, impaired hepatic citrate metabolism leads to citrate accumulation and increases T/I Ca^2+ ^ratio with normal ionized calcium [13]. Thus, citrate accumulation is indicated indirectly by an elevated T/I Ca^2+ ^ratio. Patients with hepatic or multi-organ dysfunction (or both) can develop citrate accumulation characterized by low ionized calcium, elevated total calcium, and metabolic acidosis [12,13]. In critically ill patients undergoing CRRT with regional citrate anticoagulation (CRRT-citrate), an increased T/I Ca^2+ ^ratio in about 33% of patients with severe hepatic impairment was found [13]. We prospectively evaluated the incidence and prognostic relevance of an increased T/I Ca^2+ ^ratio and its association to hepatic and multi-organ dysfunction in all patients undergoing CRRT-citrate in a medical ICU within a 2-year period.

## Materials and methods

### Patients

**Figure 1:**
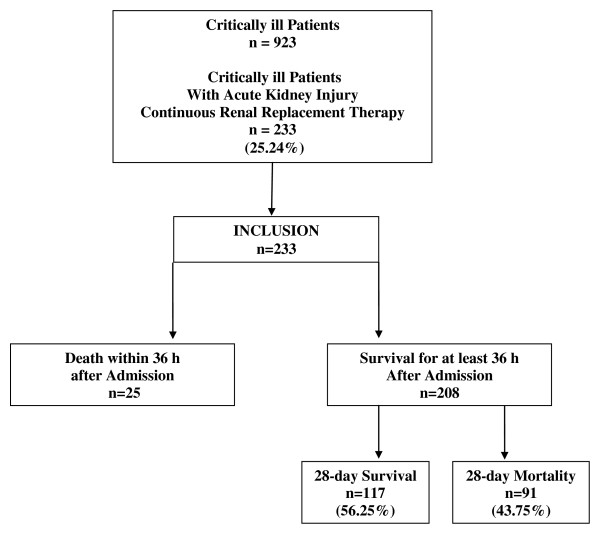
**Study flowchart for all critically ill patients admitted to the intensive care unit within a two-year period**. All patients with acute kidney injury and necessity for continuous renal replacement therapy received regional citrate anticoagulation. Twenty-three patients were excluded from analysis because hemodynamic stabilization could not be achieved within 36 hours. After hemodynamic stabilization, 208 patients were analyzed for the study.

With approval of the institutional review board (Ethical Committee of the Saarland, Germany, 211/11), we evaluated all critically ill patients with AKI and necessity for CRRT in the medical ICU of the University Hospital of Saarland from September 2009 to September 2011. Informed consent was obtained from all enrolled patients or substitute decision makers. Critical illness was defined by a commonly used score in intensive care medicine (Simplified Acute Physiology Score II, or SAPS II) [17]. Thus, critical illness and MODS were defined as a minimum SAPS II of 30 points. As shown in Figure 1, in our center, all patients with AKI and necessity for CRRT were assigned to regional citrate anticoagulation (CRRT-citrate). A steady state of calcium homeostasis was defined when a stable T/I Ca^2+ ^ratio after at least 36 hours of CRRT was achieved and when no changes in the infusion rates of CaCl_2 _or citrate were necessary in the previous 36 hours. The primary endpoint was 28-day mortality. Secondary endpoints were changes in liver function and calcium homeostasis during citrate anticoagulation.

According to the American College of Chest Physicians evidence-based clinical practice guidelines [18,19], in all patients on CRRT-citrate, unfractioned heparin (UFH) was given as an adjunctive in a thromboembolic prophylactic dose regimen (300 to 500 IU/hour intravenously). In cases of the demand for systemic effective anticoagulation (for example, deep venous thrombosis, pulmonary embolism, atrial fibrillation, mechanical heart valves, and decompensated heart failure), UFH was given in a therapeutic dosage. During the study period, in 11 patients (1.5%), a heparin-induced thrombocytopenia was diagnosed. These patients on CRRT-citrate were additionally treated with argatroban in therapeutic doses.

### Safety

Patients underwent physical examination, hemodynamic measurements (blood pressure and heart rate), and clinical laboratory tests daily. Bleeding episodes were classified according to the modified McMaster criteria [20]. The episodes were defined as minor when bleeding signs occurred but no transfusion was needed and as major when transfusion of blood or coagulation factors became necessary.

### Continuous renal replacement therapy protocol

Vascular access for CRRT was established through a double-lumen central venous catheter. Continuous venovenous hemodialysis was performed with a polysulfone high-flux hemodialyzer by using a Fresenius multifiltrate (Fresenius, Bad Homburg, Germany) with a bicarbonate dialysate at a flow rate of 2,000 mL/hour. The blood flow was 100 mL/minute. The ultrafiltration rate depended on clinical requirements (0 to 200 mL/hour). The hemodialysis tubes and filters were renewed every 72 hours. We used regional anticoagulation with 4% trisodium citrate solution (Fresenius), which was administered in the extracorporeal circuit before the site of the hemofilter. We started with a standard dose of 4 mmol/L citrate/blood. In most patients, only minor corrections in this dosing were necessary (3.5 to 4.2 mmol/L) to achieve calcium levels of 0.25 to 0.35 mmol/L in the blood of the extracorporeal circuit. On average, 4 mmol/L citrate/blood flow was used. The postfilter CaCl_2 _replacement was accomplished by CaCl_2 _infusion given via a central line before reinfusion of blood into the patient. Citrate and CaCl_2 _infusions were adjusted by the nursing team in accordance with the protocol of the manufacturer (Fresenius). The target level of intracorporeal ionized systemic calcium was between 0.9 and 1.2 mmol/L.

In cases (n = 16) of an increasing T/I Ca^2+ ^ratio of at least 2.4, which is an indirect sign of citrate accumulation, the dialysate flow was elevated up to 3,000 mL/hour in order to increase the citrate clearance. In two of these cases, as increasing dialysate flow was not efficient enough to reduce the ratio, blood flow was reduced to 80 mL/hour in order to reduce citrate offer for anticoagulation.

### Laboratory tests

Ionized postfilter extracorporeal calcium and ionized systemic calcium were measured initially every 20 minutes until a steady state was achieved and then every 4 hours. Sodium, potassium, and arterial blood gases were analyzed every 4 hours. Hemoglobin, hematocrit, red and white blood cells, platelets, creatinine, urea, total calcium, chloride, total bilirubin, albumin, cholinesterase, prothrombin time, antithrombin III, and transaminases were measured daily.

### Assessment of hepatic function

Transaminases, total bilirubin, albumin, cholinesterase, prothrombin time, and antithrombin III were measured daily. In patients with signs of progressive hepatic impairment and threatening hepatic failure, liver function was evaluated to avoid overdosage of administered therapeutics. For dynamic liver function, indocyanine green (ICG-PULSION; Pulsion Medical Systems, Munich, Germany) was injected in the central venous catheter (0.5 mg/kg). ICG is nearly exclusively eliminated by the liver unconjugatedly into the bile and does not undergo enterohepatic recirculation. Its elimination may be expressed as the plasma disappearance rate (ICG-PDR) and can be measured non-invasively at the bedside by transcutaneous pulse densitometry with a finger clip (LiMON monitoring; Pulsion Medical Systems). Normal values for ICG-PDR are considered to be more than 18% per minute. In general, ICG removal from the blood depends on liver blood flow, parenchymal cellular function, and biliary excretion [21-23]. ICG-PDR measurements were made once daily.

### Statistical analysis

Data are expressed as mean value ± standard error of the mean unless otherwise stated. All quantitative parameters were normally distributed (Kolmogorov-Smirnov test). Correlations of biomarkers were done by Pearson correlation coefficient. For quantitative data, we used one-way analysis of variance to compare three groups (T/I Ca^2+ ^ratio of less than 2.00, 2.00 to 2.39, and 2.40 or more) with Bonferroni *post hoc *test. Dependencies in contingency tables (categorical data) were analyzed with chi-squared test. Some biomarkers were used as diagnostic tests. The cutoff points were determined by the receiver operating characteristic (ROC) analysis. Survival curves were created by using the Kaplan-Meier method. Cox proportional hazards regression analysis was performed to determine predictor variables for 28-day survival. All statistical analyses were performed with StatView (SAS Institute Inc., Cary, NC, USA).

## Results

### Baseline characteristics

This prospective monocentric clinical study was performed at an interdisciplinary medical ICU. During a study period of 2 years, AKI was diagnosed in 233 of 923 critically ill patients (25.24%). In all 233 cases, CRRT-citrate was performed. A steady state of calcium homeostasis was defined when no changes in the infusion rates of CaCl_2 _or citrate were necessary within 36 hours. Consecutively, 208 patients (89.3%) were analyzed (Figure 1) and were distributed into three groups by tertiles of T/I Ca^2+ ^ratio (less than 2.00, 2.00 to 2.39, and 2.40 or more). The main demographic data are presented in Table 1. Among these three groups, there were no differences in age, gender, reasons for MODS and AKI, any history of kidney disease, and severity of critical illness as measured by SAPS II. In the highest calcium ratio tertile, patients with a history of liver disease were significantly cumulated (7.7% versus 6.7% versus 24.5%, *P *= 0.003). Laboratory parameters at the start, day 3, and last day of CRRT are depicted in Table 2.

**Table 1:** Baseline characteristics and concomitant therapies at start of continuous renal replacement therapy with regional citrate antigoagulation

Characteristic or therapy	Ratio of total to ionized Ca^2+^			ANOVA
		
	<2.0	2.0-2.39	≥2.4	or χ^2 ^test
Number	65	90	53	-
Age, years	62.6 ± 1.9	66.6 ± 1.5	62.8 ± 1.8	0.159
Females/males, number	25/40	38/52	27/26	0.382
Reasons for critical illness and MODS, number (percentage)				
Hemorrhagic shock	4 (6.2)	3 (3.3)	2 (3.8)	0.778
Septic-toxic shock	42 (61.6)	64 (71.1)	38 (71.7)	0.951
Cardiogenic shock	19 (29.2)	23 (25.6)	13 (24.5)	0.396
History of liver disease, number (percentage)				
Child B-C liver cirrhosis	5 (7.7)	6 (6.7)	13 (24.5)	0.003
History of kidney disease, number				
K/DOQI 0-I	51	67	47	0.331
K/DOQI II	2	8	1	0.123
K/DOQI III	2	2	0	0.463
K/DOQI IV	2	5	1	0.505
K/DOQI V	8	8	4	0.651
Critical illness score, hemodynamics				
SAPS II, points	46 ± 1.2	47 ± 1.1	49 ± 1.3	0.234
Heart rate, beats per minute	89 ± 2.4	90 ± 1.8	98 ± 2.7	0.021
Mean arterial pressure, mm Hg	76.3 ± 1.8	71.8 ± 1.2	68.6 ± 1.3	0.002
Concomitant therapy				
Mechanical ventilation, number (percentage)	44 (67.69)	64 (71.11)	40 (75.47)	0.650
Vasopressors, number (percentage)	44 (67.69)	64 (71.11)	40 (75.47)	0.650
Norepinephrine dose, µg/kg per minute	0.08 ± 0.05	0.10 ± 0.04	0.12 ± 0.04	<0.001
Dialysis function				
Citrate, mmol/L	3.9 ± 0.1	3.9 ± 0.1	3.8 ± 0.2	0.758
Blood flow, mL/minute	100	100	100	-
Dialysate flow, mL/hour	2,000	2,000	2,000	-
Dialysis dose, mL/kg per hour	25 ± 0.2	26 ± 0.2	25 ± 0.2	0.483
Filter patency, hours	67 ± 0.7	69 ± 0.6	67 ± 0.8	0.136
CRRT duration, days	19.7 ± 3.4	11.4 ± 1.4	8.6 ± 0.9	0.002
Reasons for CRRT termination, number (percentage)				
Restoration of renal function	62 (95.4)	55 (61.1)	0 (0)	<0.001
Death of patient	3 (4.6)	35 (38.9)	53 (100)	<0.001
Mortality, number (percentage)				
28-day mortality	3 (4.6)	35 (38.9)	53 (100)	<0.001
Hospital mortality	18 (27.7)	41 (45. 6)	53 (100)	<0.001

Data are presented as number (percentage) or as mean ± standard error of the mean. Quantitative data were analyzed by analysis of variance (ANOVA). Categorical data were analyzed by chi-squared test. CRRT, continuous renal replacement therapy; K/DOQI, Kidney Disease Outcomes Quality Initiative; MODS, multiple organ dysfunction syndrome; SAPS II, Simplified Acute Physiology Score II.

**Table 2:** Laboratory parameters at start, day 3, and last day of continuous renal replacement therapy

Parameters	T/I-Ca^2+ ^ratio									Bonferroni *post hoc *test		
	
	<2.0			2.0-2.39			≥2.4			Tertile 1 versus tertile 2	Tertile 1 versus tertile 3	Tertile 2 versus tertile 3
				
	n = 65			n = 90			n = 53					
				
	First day	Day 3	Last day				First day	Day 3	Last day			
**Critical illness score, hemodynamics**										***P *values**		

SAPS II, points	46 ± 1.21	35 ± 1.45	33 ± 1.07	47 ± 1.18	44 ± 1.38	42 ± 1.63	49 ± 1.27	59 ± 1.38	60 ± 1.41	<0.001	<0.001	<0.001
Heart rate, beats per minute	89 ± 2.37	88 ± 2.36	77 ± 2.33	90 ± 1.79	89 ± 1.82	81 ± 2.42	98 ± 2.66	97 ± 2.65	104 ± 3.07	0.324	<0.001	<0.001
MAP, mm Hg	76.34 ± 1.83	76.34 ± 1.38	79.88 ± 1.69	71.82 ± 1.15	71.71 ± 1.15	73.16 ± 1.19	68.60 ± 1.29	68.61 ± 1.30	67.28 ± 1.50	<0.001	<0.001	<0.001
Liver function												
Bilirubin, mg/dL	2.21 ± 0.66	2.28 ± 0.66	3.09 ± 0.81	2.63 ± 0.57	2.60 ± 0.58	2.99 ± 0.50	5.74 ± 1.30	6.09 ± 1.22	7.09 ± 1.25	0.799	<0.001	<0.001
Albumin, g/L	26.42 ± 0.73	25.82 ± 0.66	26.12 ± 0.72	28.11 ± 0.71	28.02 ± 0.65	27.33 ± 0.61	28.11 ± 0.86	27.42 ± 0.82	25.19 ± 0.86	0.003	0.258	0.118
CHE × 10^3^, U/L	4.52 ± 0.23	4.52 ± 0.23	4.44 ± 0.23	4.31 ± 0.20	4.29 ± 0.20	4,12 ± 0.19	3.93 ± 0.25	3.93 ± 0.25	3.85 ± 0.25	0.159	0.001	0.035
Prothrombin time, percentage	63.12 ± 3.00	72.83 ± 2.86	72.25 ± 2.79	60.12 ± 2.42	60.60 ± 2.48	61.88 ± 2.66	50.23 ± 3.12	52.59 ± 3.15	49.15 ± 3.27	<0.001	<0.001	<0.001
AT III, percentage	69.71 ± 2.18	69.70 ± 2.17	70.37 ± 2.19	68.90 ± 2.21	68.84 ± 2.21	69.09 ± 2.25	58.45 ± 3.09	58.45 ± 3.09	57.59 ± 3.11	0.471	<0.001	<0.001
ASAT, U/L	449 ± 144	298 ± 108	186 ± 95	296 ± 78	404 ± 97	520 ± 126	817 ± 304	763 ± 209	1,237 ± 379	0.360	<0.001	<0.001
ALAT, U/L	204 ± 74	163 ± 59	119 ± 56	180 ± 48	239 ± 52	251 ± 53	431 ± 174	446 ± 121	462 ± 115	0.544	0.072	<0.001
γ-GT, U/L	133 ± 18	125 ± 17	222 ± 34	144 ± 18	133 ± 15	199 ± 30	121 ± 20	129 ± 20	127 ± 19	0.519	0.072	0.188
ICG-PDR, percentage/minute	15.02 ± 0.61	18.78 ± 0.43	19.60 ± 0.58	13.93 ± 0.47	15.26 ± 0.55	16.04 ± 0.65	11.16 ± 0.64	9.26 ± 0.56	8.83 ± 0.55	<0.001	<0.001	<0.001
Dialysis function, mg/dL												
Urea	123.03 ± 8.31	63.71 ± 5.13	53.79 ± 5.01	115.48 ± 7.78	58.97 ± 3.62	47.49 ± 3.24	121.38 ± 9.22	63.83 ± 5.00	54.55 ± 5.15	0.295	0.995	0.324
Blood urea nitrogen	57.46 ± 3.88	29.75 ± 2.39	25.12 ± 2.34	53.93 3.64	27.54 ± 1.69	22.18 ± 1.51	56.48 ± 4.77	29.81 ± 2.34	25.48 ± 2.41	0.295	0.995	0.324
Creatinine	4.14 ± 0.29	2.14 ± 0.16	1.77 ± 0.16	3.14 ± 0.20	1.75 ± 0.10	1.59 ± 0.10	3.07 ± 0.28	1.88 ± 0.11	1.65 ± 0.11	0.002	0.009	0.867
Ca homeostasis and chloride												
Ca^2+ ^extracorporeal, mmol/L	0.29 ± 0.01	0.29 ± 0.01	0.29 ± 0.01	0.29 ± 0.01	0.30 ± 0.01	0.30 ± 0.01	0.29 ± 0.01	0.30 ± 0.01	0.30 ± 0.01	0.586	0.096	0.189
Total Ca^2+^, mmol/L	1.97 ± 0.03	2.07 ± 0.03	2.10 ± 0.03	2.14 ± 0.02	2.29 ± 0.03	2.25 ± 0.03	2.30 ± 0.03	2.49 ± 0.03	2.42 ± 0.05	<0.001	<0.001	<0.001
Ionized Ca^2+^, mmol/L	1.04 ± 0.02	1.11 ± 0.01	1.11 ± 0.01	1.04 ± 0.01	1.06 ± 0.02	1.05 ± 0.01	0.98 ± 0.02	0.99 ± 0.01	0.98 ± 0.02	<0.001	<0.001	<0.001
T/I-Ca^2+ ^ratio	1.90 ± 0.02	1.87 ± 0.01	1.89 ± 0.02	2.07 ± 0.02	2.16 ± 0.01	2.15 ± 0.02	2.36 ± 0.04	2.53 ± 0.02	2.49 ± 0.04	<0.001	<0.001	<0.001
Chloride, mmol/L	108 ± 0.45	107 ± 0.58	107 ± 0.62	108 ± 0.51	108 ± 0.78	107 ± 0.54	108 ± 0,36	107 ± 0.55	108 ± 0.34	0.587	0.781	0.672
Acid-base balance												
pH	7.38 ± 0.01	7.43 ± 0.01	7.43 ± 0.01	7.40 ± 0.01	7.43 ± 0.01	7.42 ± 0.01	7.37 ± 0.01	7.41 ± 0.01	7.39 ± 0.01	0.384	<0.001	<0.001
pCO_2_, mm Hg	38.77 ± 1.48	37.81 ± 0.78	38.09 ± 0.72	35.59 ± 0.69	37.61 ± 0.60	39.25 ± 0.69	35.81 ± 0.98	37.98 ± 0.86	38.59 ± 1.22	0.242	0.301	0.994
HCO_3_, mm Hg	22.77 ± 0.53	25.10 ± 0.36	25.61 ± 0.54	22.41 ± 0.38	24,34 ± 0.36	25.28 ± 0.46	20.68 ± 0.42	23.13 ± 0.54	23.40 ± 0.67	0.273	<0.001	<0.001
Hematology												
Hemoglobin, g/dL	10.29 ± 0.26	9.24 ± 0.22	9.24 ± 0.22	10.30 ± 0.19	9.98 ± 0.27	9.98 ± 0.27	10.14 ± 0.28	9.56 ± 0.25	9.54 ± 0.25	0.009	0.495	0.081
Platelets, 10^9^/L	164 ± 10.67	147 ± 9.51	152 ± 10.99	147 ± 8.96	135 ± 7.94	127 ± 8.22	122 ± 12.88	101 ± 12.18	77 ± 10.58	0.025	<0.001	<0.001

Data are presented as mean ± standard error of the mean. Quantitative data were analyzed by Bonferroni *post hoc *test. γ-GT, gamma-glutamylcyclotransferase; ALAT, alanine aminotransferase; ASAT, aspartate aminotransferase; AT III, antithrombin III; CHE, cholinesterase; HCO_3_, bicarbonate; ICG-PDR, indocyanine green plasma disappearance rate; MAP, mean arterial pressure; pCO_2_, carbon dioxide partial pressure; SAPS II, Simplified Acute Physiology Score II; T/I-Ca^2+ ^ratio, total-to-ionized calcium ratio.

### Outcomes

**Figure 2:**
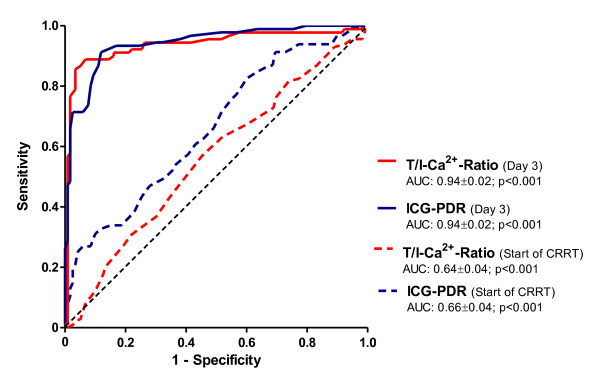
**Receiver operating characteristic curves for T/I-Ca^2+^-ratio and ICG-PDR**. AUC: area under the curve, T/I Ca^2+ ^ratio: total-to-ionized calcium ratio, ICG-PDR: indocyanine green plasma disappearance rate.

**Figure 3:**
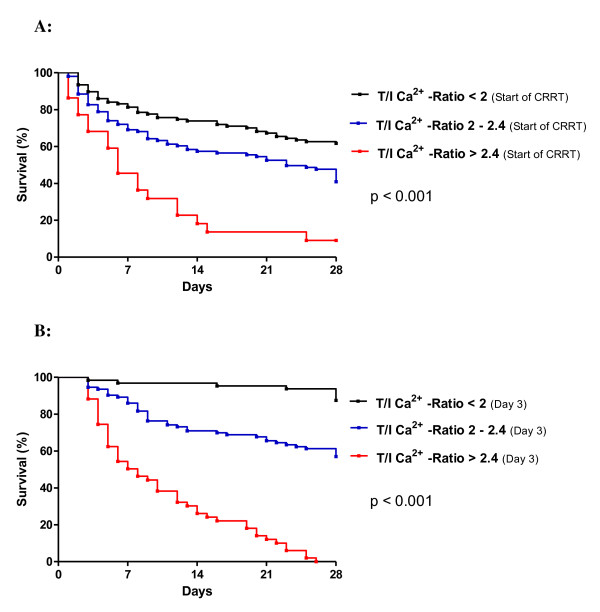
**Kaplan-Meier survival analysis using T/I Ca^2+ ^ratio at start (a) and on day 3 (b)**. A Log-rank test (p-value) was performed.

Higher T/I Ca^2+ ^ratio was associated with a significant increase in 28-day mortality (4.6% versus 38.9% versus 100%, *P *<0.001) (Table 1). Cox regression analysis, including age, gender, heart rate, mean arterial blood pressure, bilirubin, albumin, cholinesterase acticity, prothrombin time index, ICG clearance, acid-base balance, and total and ionized calcium levels and their ratio, was performed. Only albumin (*P *<0.001), ICG clearance (*P *<0.001), and total and ionized calcium levels (*P *<0.001) and their ratio (*P *<0.004) were significant independent predictors for 28-day mortality. ROC analysis, calculating the area under the curve (AUC), showed a high accuracy in predicting mortality for the T/I Ca^2+ ^ratio (AUC of 0.938 ± 0.02, *P *<0.001) at day 3. This accuracy was as high as the predictability of the well-established ICG clearance (AUC of 0.941 ± 0.02, *P *<0.001) (Figure 2). In ROC analysis, a cutoff for the T/I Ca^2+ ^ratio of at least 2.4 had a prognostic sensitivity of 55.6% (95% confidence interval (CI) 44.7% to 66.0%) and a specificity of 99.2% (95% CI 95.4% to 99.9%) to determine 28-day mortality. In the Kaplan-Meier survival analysis, patients with a ratio of at least 2.4 had the highest mortality rates (Figure 3a: baseline; Figure 3b: levels on day 3). The log-rank tests for all of these survival curves were significantly different (*P *<0.001). The hazard ratio for mortality was 33.5-fold higher for a T/I Ca^2+ ^ratio of at least 2.4 than for a ratio of less than 2.4 (Table 3).

**Table 3:** Hazard ratio for 28-day mortality

	Hazard ratio	95% confidence interval	*P *value
Total-to-ionized Ca^2+ ^ratio (day 3)			
<2 versus ≥2	4.17	2.76-6.29	<0.001
<2.2 versus ≥2.2	25.13	15.49-40.77	<0.001
<2.4 versus ≥2.4	33.50	18.18-61.71	<0.001
ICG-PDR (day 3)			
<16 versus ≥16	10.93	7.09-16.84	<0.001
<12 versus ≥12	28.63	16.41-49.94	<0.001
<8 versus ≥8	26.09	12.44-54.72	<0.001

ICG-PDR, indocyanine green plasma disappearance rate.

### Total-to-ionized calcium ratio and hepatic or multi-organ dysfunction or both

**Figure 4:**
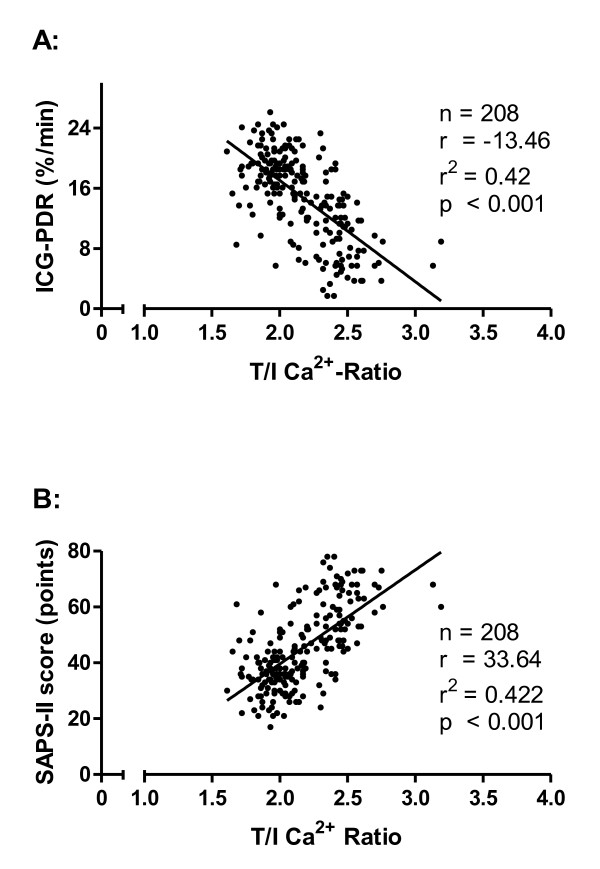
**Total-to-ionized calcium ratio (T/I Ca^2+ ^ratio) and hepatic or multi-organ dysfunction or both**. Correlation between the T/I Ca^2+ ^ratio and hepatic clearance measured by the indocyanine green plasma disappearance rate (ICG-PDR) **(a) **and multi-organ dysfunction measured by Simplified Acute Physiology Score II (SAPS II) **(b) **on day 3 of continuous renal replacement therapy with regional citrate antigoagulation. A Cox regression analysis was performed.

None of the patients on CRRT-citrate developed relevant signs of hypo- or hypercalcemia, chloride overload because of CaCl_2 _infusion, or any signs of citrate toxicity. Mean pH values, carbon dioxide partial pressure (pCO_2_), and bicarbonate levels remained in a normal range during the whole study period (Table 2). Nevertheless, according to the distribution of patients in the predefined T/I Ca^2+ ^ratio tertiles after achieving a steady state of calcium homeostasis after 72 hours, total calcium concentrations and T/I Ca^2+ ^ratios were already elevated at baseline in tertile 3 (*P *<0.001) (Table 2). Patients in tertile 3 were associated with a significantly decreased hepatic clearance, measured by the ICG-PDR. This contrasted to patients in tertiles 1 and 2 in whom hepatic clearance increased significantly (*P *<0.001) (Table 2). Strikingly, according to the duration of CRRT (baseline, day 3, and last day), T/I Ca^2+ ^ratios and ICG-PDR clearances had a strong inverse correlation (r^2 ^= 0.42, *P *<0.001) (Figure 4a). From the static liver function parameters, bilirubin trended toward higher levels and prothrombin time index was significantly reduced (*P *<0.001) in tertile 3, whereas albumin and cholinesterasis did not differ significantly between the tertiles. Thus, monitoring of liver enzymes and hepatic protein synthesis is not sensitive enough to detect functional metabolic hepatic impairment. Within the T/I Ca^2+ ^ratio tertiles, the severity of critical illness changed. Although SAPS II decreased in tertiles 1 and 2, we observed a time-dependent significant increase of SAPS II in tertile 3 (*P *<0.001) (Table 2). We found a significant correlation between the T/I Ca^2+ ^ratios and the SAPS II (r^2 ^= 0.42, *P *<0.001) (Figure 4b).

### Dialysis therapy, efficacy, and safety

CRRT-citrate was performed with a blood flow of 100 mL/hour, a dialysate flow of 2,000 mL/hour, and a dialysis dose (adjusted by patient weight) of 25 ± 0.2 versus 26 ± 0.2 versus 25 ± 0.3 mL/kg per hour (*P *= 0.483) (Table 1). In 16 of 53 patients (30.2%) with an elevated T/I Ca^2+ ^ratio of at least 2.4, dialysate flow was raised up to 3,000 mL/hour in order to increase the citrate clearance; if necessary, as a second step, blood flow was raised down to 80 mL/hour in order to reduce citrate offer for anticoagulation. These procedures were effective, avoiding a further increase of T/I Ca^2+ ^ratio and citrate accumulation. CRRT-citrate was effective for all patient groups, as evidenced by the average fall in urea, blood urea nitrogen, and creatinine (*P *<0.001) (Table 2). After achieving a steady state and comparing these parameters on the last day of therapy, they did not differ significantly. The hemodialysis tubes and filters were renewed every 67 ± 0.7 versus 69 ± 0.6 versus 67 ± 0.8 hours (*P *= 0.136) (Table 1). Fifty-five filters (5.3%) were substituted because of technical problems and vascular access malfunction. No patient developed severe bleeding.

## Discussion

CRRT-citrate is effective and safe, particularly in critically ill patients with elevated bleeding risks [7,9,10,24]. In cases of hepatic or multi-organ dysfunction or both, citrate metabolism can be impaired, resulting in citrate accumulation detected indirectly by an elevated T/I Ca^2+ ^ratio [9,13]. So far, the incidence and prognostic relevance of an elevated T/I Ca^2+ ^ratio on CRRT-citrate patients remain unclear. We prospectively evaluated the prognostic relevance of the T/I Ca^2+ ^ratio in all critically ill patients with AKI undergoing CRRT-citrate in a medical ICU within a 2-year period. We demonstrate, for the first time, that the T/I Ca^2+ ^ratio (a) emerged as an independent predictor of 28-day mortality in critically ill patients on CRRT-citrate and was associated (b) with hepatic impairment and (c) with multi-organ dysfunction.

Monitoring the T/I Ca^2+ ^ratio in patients on CRRT-citrate is an easy procedure with high therapeutic and prognostic impact. As shown in our study, measuring of total and ionized calcium levels and calculating their ratio provided independent predictors for 28-day mortality in patients on CRRT-citrate. A T/I Ca^2+ ^ratio of at least 2.4 had a prognostic sensitivity of 55.6% and a specificity of 99.2% to determine 28-day mortality. In the Kaplan-Meier survival analysis, patients with a ratio of at least 2.4 had the highest mortality rates; in these patients, the risk to die was 33.5-fold higher than in patients with a ratio of less than 2.4. Larger studies may be required to define the cutoff point with the greatest diagnostic and prognostic efficiency more precisely.

It remains unclear whether changes in calcium homeostasis are only markers of critical illness and comorbidity or whether they independently contribute to mortality. First of all, ionized calcium levels remained within the normal range by continuous CaCl_2 _infusion and are unlikely to influence patient outcomes. Furthermore, serum chloride levels were not changed. Only extreme abnormalities of ionized calcium were reported to be independent predictors of mortality [25,26]. Second, total calcium levels were increased in cases of citrate accumulation caused by hepatic or multi-organ dysfunction or both. In acute stressor states, increased total calcium levels cause a shift of calcium from the circulating pool to intracellular compartments and facilitate an intracellular calcium overload [27], inducing oxidative stress, permeability, and apoptotic cell death [26]. Furthermore, calcium administration increased mortality in animal models of sepsis [28,29].

Therefore, therapeutic strategies target the early detection of either elevated total calcium levels or elevated T/I Ca^2+ ^ratio caused by citrate accumulation in patients on CRRT-citrate. Citrate levels could be decreased by increasing citrate clearance through the use of a higher dialysate flow, by reducing the blood flow (resulting in a reduced need for citrate anticoagulation), or by reducing the citrate offer in the extracorporeal circuit. Under these strategies, the use of a higher dialysate flow (2,000 to 3,000 mL/hour) is associated with increased diffusion mechanisms and is very effective in citrate clearance [30]. Reducing blood flow by 20% (from 100 to 80 mL/hour) is associated with a decreased need of citrate for anticoagulation, but its effectiveness in lowering citrate levels is less compared with increasing dialysate flow [30]. Lowering citrate offer by reducing the citrate dose (3.5 to 4.2 mmoL/L) in the extracorporeal circuit is associated with an increased risk for coagulopathy and is recommended only in selected patients according to safety and filter patency criteria.

In critically ill patients on CRRT-citrate, total calcium levels and the T/I Ca^2+ ^ratio were increased in cases of citrate accumulation. This decreased citrate metabolism can be caused by a hepatic or multi-organ dysfunction or both. We found a surprisingly high incidence of an increased T/I Ca^2+ ^ratio: close to 25%. Meier-Kriesche and colleagues [13] described an incidence of 12%. Another retrospective cohort study found, in patients with hepatic impairment, lower ionized calcium levels without elevated T/I Ca^2+ ^ratio [9]. Neither study reported the severity of critical illness.

Monitoring liver enzymes and hepatic protein synthesis has only limited diagnostic sensitivity for hepatic dysfunction [31,32]. The dynamic hepatic clearance test, the indocyanine green plasma disappearance rate (ICG-PDR), is a valuable tool for sensitive and specific assessment of liver function and is shown to correlate with outcomes in critically ill patients [21-23]. Our data demonstrate a significant inverse correlation between hepatic function measured by the ICG-PDR and the T/I Ca^2+ ^ratio in patients on CRRT-citrate. Although an increase in the T/I Ca^2+ ^ratio was observed, critical levels were not achieved. Thus, hepatic impairment does not represent a contraindication for the use of regional anticoagulation with citrate. Elevated T/I Ca^2+ ^ratios were also associated with the severity of critically illness, as measured by SAPS II. This could be caused by poor tissue perfusion because citrate metabolism takes place in tissues rich in mitochondria, such as liver, skeletal muscles, and kidney. Therefore, a very close monitoring of calcium homeostasis in patients with hepatic impairment and MODS is strongly recommended to detect early citrate accumulation.

## Conclusions

In patients on CRRT-citrate, T/I Ca^2+ ^ratio is closely related to the clinical outcome and emerged as an independent predictor of 28-day mortality. Larger studies are required to define the cutoff and predictive value for the T/I Ca^2+ ^ratio. This ratio is associated with hepatic or multi-organ dysfunction or both and therefore is an important therapeutic target.

## Key messages

In critically ill patients on CRRT-citrate, an elevated T/I Ca^2+ ^ratio

• is an independent predictor for 28-day mortality,

• is associated with hepatic or multi-organ dysfunction or both,

• is an indirect marker of systemic citrate accumulation, and

• signals the necessity to increase citrate clearance.

## Abbreviations

AKI: acute kidney injury; AUC: area under the curve; CaCl_2_: calcium chloride; CI: confidence interval; CRRT: continuous renal replacement therapy; CRRT-citrate: continuous renal replacement therapy with regional citrate antigoagulation; ICG: indocyanine green; ICG-PDR: indocyanine green plasma disappearance rate; ICU: intensive care unit; MODS: multiple organ dysfunction syndrome; ROC: receiver operating characteristic; SAPS II: Simplified Acute Physiology Score II; T/I Ca^2+ ^ratio: total-to-ionized calcium ratio; UFH: unfractioned heparin.

## Competing interests

The authors declare that they have no competing interests.

## Authors' contributions

AL helped to initiate the study, to perform CRRT, and to conduct all the investigations and the statistical analysis of the data and drafted the manuscript. MK helped to initiate the study and to administer CRRT. AL-G and DF helped to administer CRRT. TS was responsible for data management. RR, JP, and MB helped to conduct all the investigations and the statistical analysis of the data. All authors participated in interpreting the data and read and approved the final manuscript.

## References

[B1] Bellomo R Kellum J Ronco C Acute renal failure: time for consensus Intensive Care Med 2001 27 1685 1688 10.1007/s00134-001-1120-6 11810109

[B2] Bagshaw SM Uchino S Bellomo R Morimatsu H Morgera S Schetz M Tan I Bouman C Macedo E Gibney N Tolwani A Oudemans-van Straaten HM Ronco C Kellum JA Septic acute kidney injury in critically ill patients: clinical characteristics and outcomes Clin J Am Soc Nephrol 2007 2 431 439 10.2215/CJN.03681106 17699448

[B3] Uchino S The epidemiology of acute renal failure in the world Curr Opin Crit Care 2006 12 538 543 10.1097/01.ccx.0000247448.94252.5a 17077683

[B4] Tonelli M Manns B Feller-Kopman D Acute renal failure in the intensive care unit: a systematic review of the impact of dialytic modality on mortality and renal recovery Am J Kidney Dis 2002 40 875 885 10.1053/ajkd.2002.36318 12407631

[B5] Vinsonneau C Camus C Combes A Costa de Beauregard MA Klouche K Boulain T Pallot JL Chiche JD Taupin P Landais P Dhainaut JF Continuous venovenous haemodiafiltration versus intermittent haemodialysis for acute renal failure in patients with multiple-organ dysfunction syndrome: a multicentre randomised trial Lancet 2006 368 379 385 10.1016/S0140-6736(06)69111-3 16876666

[B6] Ronco C Continuous dialysis is superior to intermittent dialysis in acute kidney injury of the critically ill patient Nat Clin Pract Nephrol 2007 3 118 119 10.1038/ncpneph0423 17322924

[B7] Oudemans-van Straaten HM Wester JP de Pont AC Schetz MR Anticoagulation strategies in continuous renal replacement therapy: can the choice be evidence based? Intensive Care Med 2006 32 188 202 10.1007/s00134-005-0044-y 16453140

[B8] Tolwani AJ Wille KM Anticoagulation for continuous renal replacement therapy Semin Dial 2009 22 141 145 10.1111/j.1525-139X.2008.00545.x 19426417

[B9] Durao MS Monte JC Batista MC Oliveira M Iizuka IJ Santos BF Pereira VG Cendoroglo M Santos OF The use of regional citrate anticoagulation for continuous venovenous hemodiafiltration in acute kidney injury Crit Care Med 2008 36 3024 3029 10.1097/CCM.0b013e31818b9100 18824904

[B10] Oudemans-van Straaten HM Bosman RJ Koopmans M van der Voort PH Wester JP van der Spoel JI Dijksman LM Zandstra DF Citrate anticoagulation for continuous venovenous hemofiltration Crit Care Med 2009 37 545 552 10.1097/CCM.0b013e3181953c5e 19114912

[B11] Tolwani AJ Palevsky PM Introduction. The clinical application of CRRT--current status Semin Dial 2009 22 107 10.1111/j.1525-139X.2008.00557.x 19426411

[B12] Kramer L Bauer E Joukhadar C Strobl W Gendo A Madl C Gangl A Citrate pharmacokinetics and metabolism in cirrhotic and noncirrhotic critically ill patients Crit Care Med 2003 31 2450 2455 10.1097/01.CCM.0000084871.76568.E6 14530750

[B13] Meier-Kriesche HU Gitomer J Finkel K DuBose T Increased total to ionized calcium ratio during continuous venovenous hemodialysis with regional citrate anticoagulation Crit Care Med 2001 29 748 752 10.1097/00003246-200104000-00010 11373461

[B14] Nowak MA Campbell TE Profound hypercalcemia in continuous veno-venous hemofiltration dialysis with trisodium citrate anticoagulation and hepatic failure Clin Chem 1997 43 412 413 9023154

[B15] Cote CJ Goldstein EA Fuchsman WH Hoaglin DC The effect of nail polish on pulse oximetry Anesth Analg 1988 67 683 686 3382042

[B16] Díaz J Acosta F Parrilla P Sansano T Contreras RF Bueno FS Martínez P Correlation among ionized calcium, citrate, and total calcium levels during hepatic transplantation Clin Biochem 1995 28 315 317 10.1016/0009-9120(94)00094-C 7554253

[B17] Le Gall JR Lemeshow S Saulnier F A new Simplified Acute Physiology Score (SAPS II) based on a European/North American multicenter study JAMA 1993 270 2957 2963 10.1001/jama.1993.03510240069035 8254858

[B18] Geerts W Selby R Prevention of venous thromboembolism in the ICU Chest 2003 124 357S 363S 10.1378/chest.124.6_suppl.357S 14668418

[B19] Geerts WH Bergqvist D Pineo GF Heit JA Samama CM Lassen MR Colwell CW Prevention of venous thromboembolism: American College of Chest Physicians Evidence-Based Clinical Practice Guidelines (8th Edition) Chest 2008 133 381S 453S 10.1378/chest.08-0656 18574271

[B20] Graafsma YP Prins MH Lensing AW de Haan RJ Huisman MV Buller HR Bleeding classification in clinical trials: observer variability and clinical relevance Thromb Haemost 1997 78 1189 1192 9364983

[B21] Kimura S Yoshioka T Shibuya M Sakano T Tanaka R Matsuyama S Indocyanine green elimination rate detects hepatocellular dysfunction early in septic shock and correlates with survival Crit Care Med 2001 29 1159 1163 10.1097/00003246-200106000-00014 11395594

[B22] Sakka SG Assessing liver function Curr Opin Crit Care 2007 13 207 214 10.1097/MCC.0b013e328012b268 17327744

[B23] Sakka SG Reinhart K Wegscheider K Meier-Hellmann A Comparison of cardiac output and circulatory blood volumes by transpulmonary thermo-dye dilution and transcutaneous indocyanine green measurement in critically ill patients Chest 2002 121 559 565 10.1378/chest.121.2.559 11834672

[B24] Mehta RL McDonald BR Aguilar MM Ward DM Regional citrate anticoagulation for continuous arteriovenous hemodialysis in critically ill patients Kidney Int 1990 38 976 981 10.1038/ki.1990.300 2266683

[B25] Egi M Kim I Nichol A Stachowski E French CJ Hart GK Hegarty C Bailey M Bellomo R Ionized calcium concentration and outcome in critical illness Crit Care Med 2011 39 314 321 10.1097/CCM.0b013e3181ffe23e 21099425

[B26] Hadique S Khamare C Finkel MS The Frog Prince of calcium homeostasis Crit Care Med 2011 39 406 408 10.1097/CCM.0b013e318205c34d 21248524

[B27] Whitted AD Stanifer JW Dube P Borkowski BJ Yusuf J Komolafe BO Davis RC Jr Soberman JE Weber KT A dyshomeostasis of electrolytes and trace elements in acute stressor states: impact on the heart Am J Med Sci 2010 340 48 53 10.1097/MAJ.0b013e3181e5945b 20610973

[B28] Malcolm DS Zaloga GP Holaday JW Calcium administration increases the mortality of endotoxic shock in rats Crit Care Med 1989 17 900 903 10.1097/00003246-198909000-00012 2504540

[B29] Zaloga GP Sager A Black KW Prielipp R Low dose calcium administration increases mortality during septic peritonitis in rats Circ Shock 1992 37 226 229 1423913

[B30] Swartz R Pasko D O'Toole J Starmann B Improving the delivery of continuous renal replacement therapy using regional citrate anticoagulation Clin Nephrol 2004 61 134 143 14989634 10.5414/cnp61134

[B31] Reichling JJ Kaplan MM Clinical use of serum enzymes in liver disease Dig Dis Sci 1988 33 1601 1614 10.1007/BF01535953 2904353

[B32] Shakil AO Kramer D Mazariegos GV Fung JJ Rakela J Acute liver failure: clinical features, outcome analysis, and applicability of prognostic criteria Liver Transpl 2000 6 163 169 10719014 10.1002/lt.500060218

